# Surgical management of post-circumcision webbed penis in children

**DOI:** 10.1080/2090598X.2020.1722518

**Published:** 2020-02-09

**Authors:** Mohamed A. Negm, Salah A. Nagla

**Affiliations:** a Pediatric Surgery Unit, Qena Faculty of Medicine, South Valley University, Qena, Egypt; b Urology Department, Faculty of Medicine, Tanta University, Tanta, Egypt

**Keywords:** Circumcision, webbed penis, scrotoplasty

## Abstract

**Objective:**

To report the outcomes of surgical correction of post-circumcision webbed penis using two previously described techniques: the Heineke-Mikulicz (HM) scrotoplasty and the multiple Z-plasty.

**Patients and methods:**

A prospective study of children with post-circumcision webbed penis was conducted. The patients were classified into two groups according to the degree of web and the remaining ventral penile skin as to whether adequate or short after circumcision. Group I was repaired by HM scrotoplasty and in Group II the multiple Z-plasty technique was used.

**Results:**

This study included 86 patients of whom 71 maintained follow-up; 44 (62%) in Group I and 27 (38%) in Group II. The median (range) operative time was 45 (30–55) min in Group I and 75 (60–90) min in Group II. Wound infection occurred in two (4.5%) patients in Group I. In Group II postoperative mild self-limited penile oedema was present in three patients (11.1%). A self-limited scrotal haematoma developed in two (7.4%) patients.

**Conclusion:**

Correction of post-circumcision webbed penis in children can be done by one of two techniques: HM scrotoplasty in Grade 1 and multiple Z-plasty in Grade 2 and Grade 3, with favourable outcomes.

**Abbreviations:**

HM: Heineke-Mikulicz; IQR: interquartile range

## Introduction

Webbed penis is a condition in which a skin fold tethers the scrotum to the ventral penile shaft obscuring the penoscrotal angle [1]. This anomaly is usually discovered in infancy or at circumcision. In our locality, circumcision is done on a ritual and religious basis in almost every male. It is sometimes done by non-medical practitioners; hence, the discovery of such an anomaly may be missed. Correction of this web after circumcision is not an easy procedure due to the loss of the preputial tissue. Many studies have been conducted on the correction of primary webbed penis [2,3]. To our knowledge, no study has been published solely on the correction of post-circumcision webbed penis. Thus, the aim of the present study was to propose surgical correction techniques for different grades of post-circumcision webbed penis by using the Heineke-Mikulicz (HM) scrotoplasty and multiple Z-plasty techniques in children.

## Patients and methods

This prospective study included 86 children, from April 2014 to March 2019, in two centres (Level III evidence), who presented to our departments with post-circumcision webbed penis. The study began after obtaining Ethics Committee approval. Before surgery, a detailed consent was signed by the guardians of the children after explaining the hazards and possible complications of the procedures. Moreover, this study followed the principles of the Declaration of Helsinki.

Inclusion criteria:

- First discovered webbed penis in circumcised child.
- All grades of webbed circumcised penis.
- Follow-up of ≥6 months after web correction.

Exclusion criteria:

- Previously corrected webbed penis.
- Webbed circumcised penis with both deficient ventral and dorsal skin.
- History of penile and/or scrotal wounds, which mandated surgical repair.

**Figure 1.**
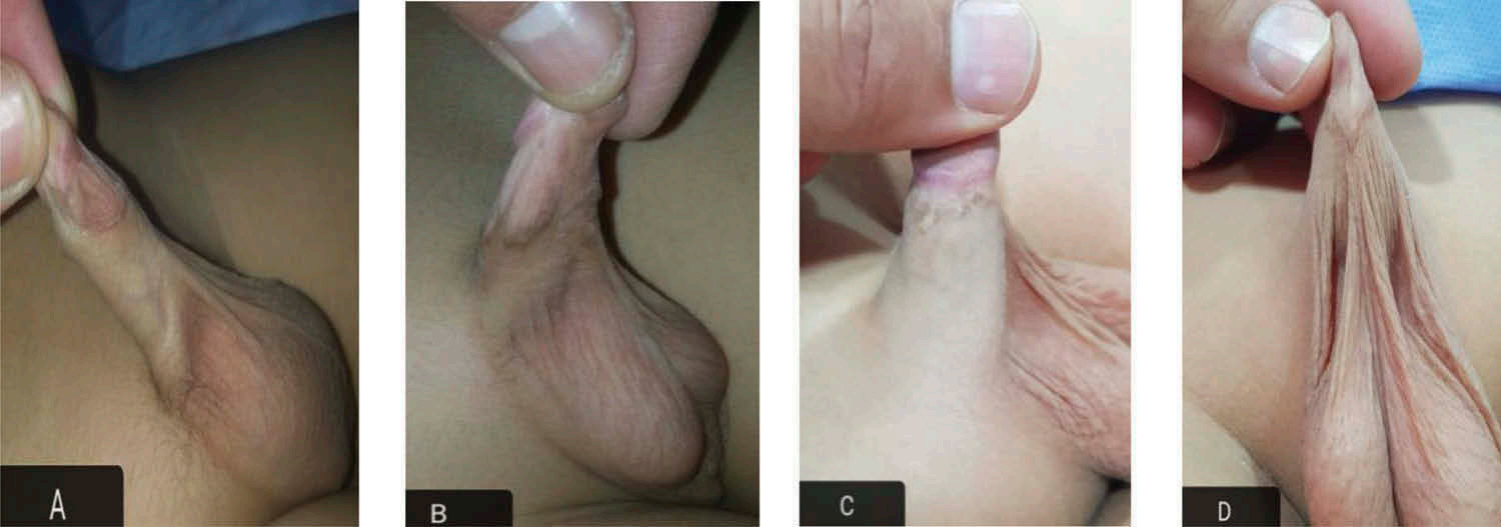
The web attached to penile shaft at: (a) proximal third, (b) middle third, (c) and distal third, (d) Broad web to distal third.

After local examination, the patients were graded into three grades according to the El Koutby and El Gohary [4] classification of primary webbed penis and the condition of the ventral penile skin (Figure 1). They were later divided into two groups:

- Group I: Included patients with Grade 1 webbed penis with adequate ventral skin.
- Group II: Included patients with Grade 2 and 3 webbed penis with deficient ventral skin.

Two techniques were utilised for correction of post-circumcision webbed penis: the HM scrotoplasty for Group I and the multiple Z-plasty for Group II.

### Procedures

**Figure 2.**
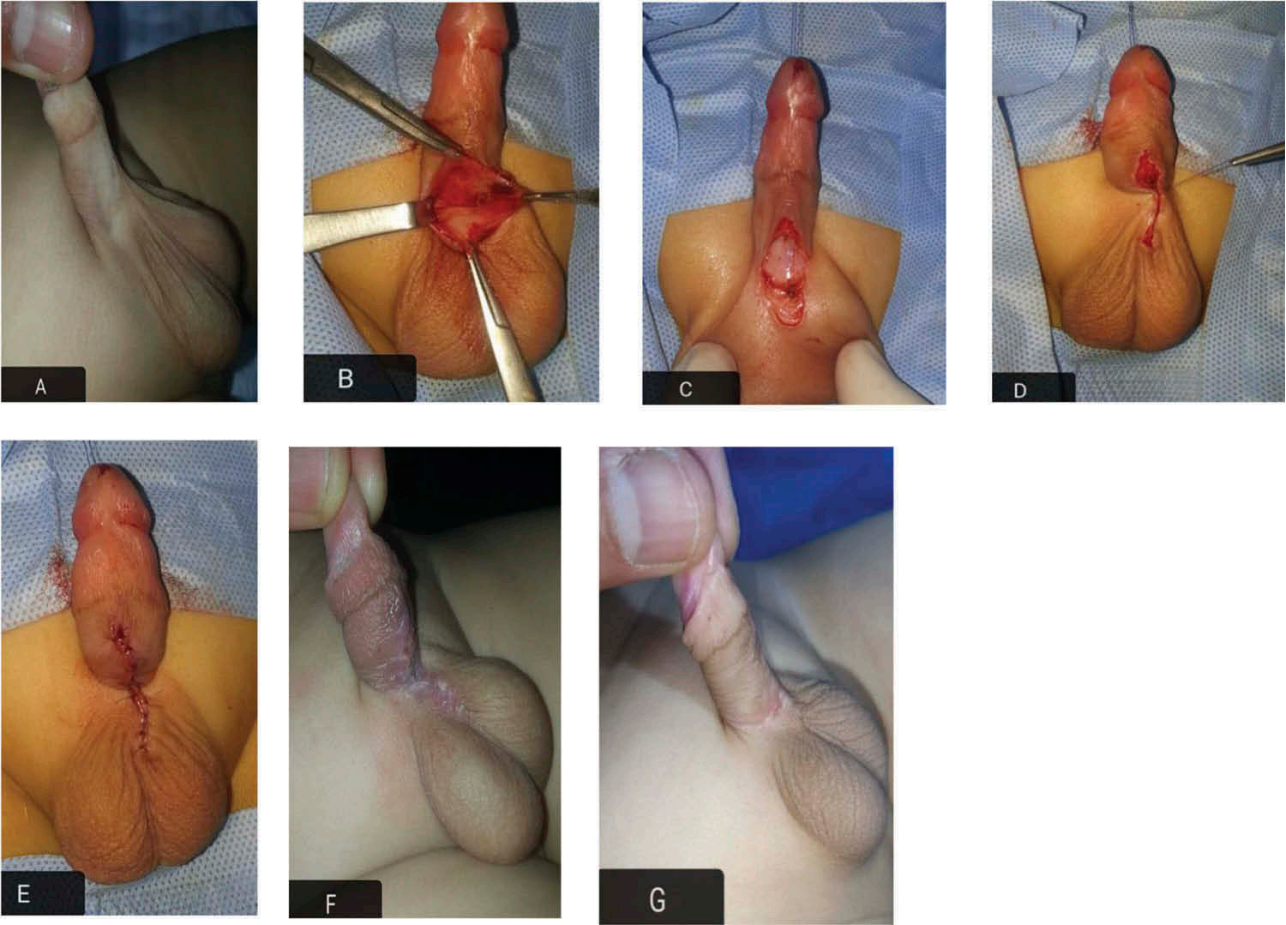
HM scrotoplasty technique. (a) preoperative, (b) transverse incision at penoscrotal junction, (c) after separation of the scrotal dartos from the penile dartos, (d) skin closure, (e) vertical closure with web correction, (f) 2 weeks later, and (g) at the 6-month follow-up.

**Figure 3.**
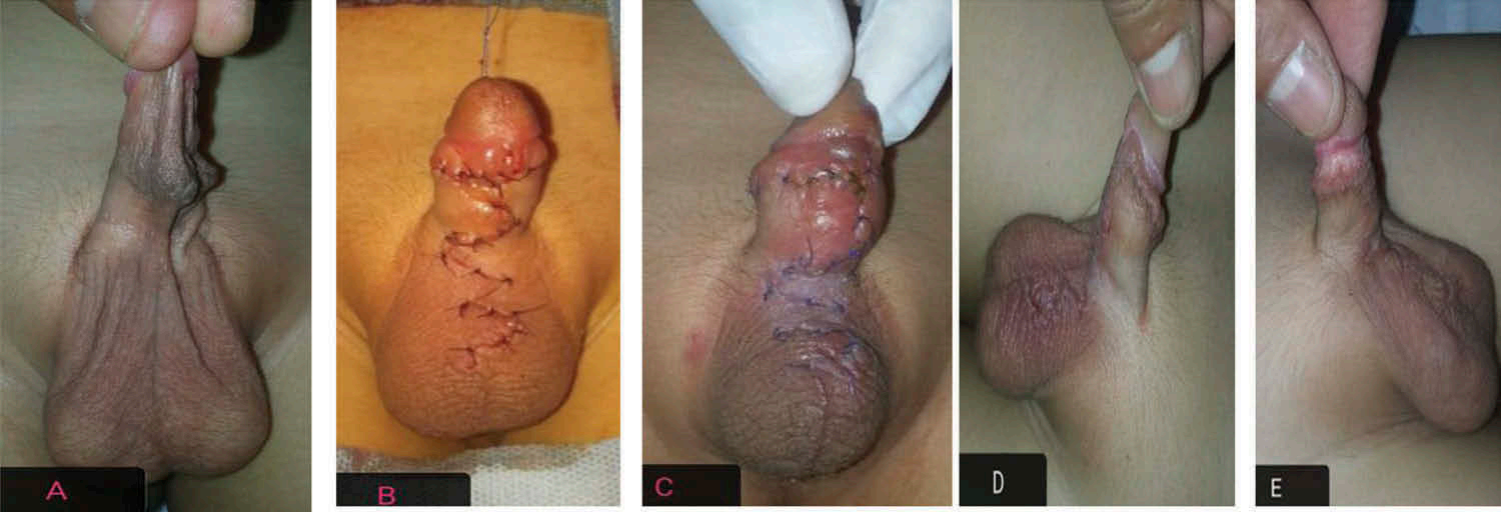
Multiple Z-plasty technique. (a) preoperative, (b) immediately postoperatively, (c) 2 weeks later, (d) after 2 months, and (e) at the 6-month follow-up.

In all cases, preoperative parenteral antibiotic was given according to the weight of the child. General intubated anaesthesia was used supplemented by caudal analgesia. In Group I, a glanular traction suture was taken (Figure 2). The web was identified at the junction of penile ventral skin (smooth) from the scrotal skin (corrugated). At the penoscrotal web, a transverse incision was made. The incision was deepened to separate the scrotal dartos fascia from its attachment to the penile dartos fascia. This incision was closed vertically by approximation of the dartos fascia and the skin with 6/0 polyglactin 910 (Vicryl®; Ethicon Inc., Somerville, NJ, USA) interrupted sutures. In Group II, the technique started with penile degloving with a circumcising incision 2 mm proximal to the corona. Degloving reached the penoscrotal junction to remove any abnormal dartos attachments. Multiple Z-plasty incisions were drawn with wide-based flaps to avoid necrosis (two or three according to ventral skin deficiency) at the penoscrotal junction to lengthen the ventral skin. We approximated the underneath fascia and closed the skin with 6\0 polyglactin 910 (Figure 3). A simple gauze penile dressing was applied for 3–4 days without any urinary diversion. All the patients were discharged on the same day as the operation. Oral antibiotic was prescribed according to the child’s weight for 4–5 days postoperatively. The patients were followed-up at 2 weeks, 6 months, and annually thereafter. The primary outcome was the disappearance of the penile web. The secondary outcomes included scrotal haematoma, wound infection, wound disruption, penile oedema, newly developed and/or residual penile curvature and penile torsion. Moreover, the type of scar healing was checked at ≥6 months. The statistical analysis was done by an independent statistician using the Statistical Package for the Social Sciences (SPSS®), version 20 (SPSS Inc., IBM Corp., Armonk, NY, USA). We used descriptive statistics in the form of median, range, minimum and maximum, and interquartile range (IQR) for non-parametric data.

## Results

This study included 86 patients but only 71 completed the study; 44 (62%) in Group I and 27 (38%) in Group II (17 patients were Grade 2 and 10 were Grade 3). The presenting complaint was small sized penis in 46 (64.8%) patients, complications of circumcision in 21 (29.6%), and hidden penis in four (5.6%) (Table 1). Circumcision was done by non-medical personnel in 34 (47.9%) patients and a non-paediatric surgeon in 37 (52.1%). The age at circumcision in both groups ranged from 1–8 months. Their ages at surgery ranged from 6–72 months in both groups. The operative time was significantly longer in Group II, ranging from 40 to 90 min vs 30–60 min in Group I (Table 2). In Group I, wound infection occurred in two (4.5%) patients. In Group II, postoperative mild self-limited penile oedema was present in three (11.1%) patients. Only one patient developed superficial wound dehiscence that needed daily dressing. A self-limited scrotal haematoma was present in two (7.4%) patients. Two patients in Group II still had Grade 1 webbed penis but, the parents were satisfied with the results and none of the studied cases needed revision surgery. Although complications were encountered in 11.3%, the success rate of achieving the primary outcome was 97%. The follow-up period was ≥6 months.

**Table 1.** Patients’ presentation.

Presentation	Number of patients (%)
Small-sized penis	46 (64.8)
Complications after circumcision Ugly scar Glandular synechiae Meatal stenosis Smegmoma	21(29.6) 10 4 4 3
Hidden penis	4 (5.6)

**Table 2.** Personal, operative and postoperative data in both groups.

Variable, median (IQR)	Group I HM scrotoplasty	Group II Z-plasty
Age at circumcision, months	3 (2–4)	3 (2–4)
Age at operation, months	36 (18–49.5)	35 (24–50)
Operation time, min	45 (40–50)	75 (60–85)
Follow-up, months	16 (10.25–30.5)	13 (8–24)

IQR, 25th to 75th percentiles.

## Discussion

Circumcision is a common procedure that is greatly affected by cultural and religious traditions. It is estimated that one-third of males worldwide are circumcised, with a high prevalence in some countries [5,6]. In our country, virtually all males are circumcised for ritual and religious reasons. The presentation of penile anomalies, such as penoscrotal web after circumcision, may be due to improper assessment before circumcision or because of the circumcision being performed by an inexpert surgeon or even a non-medical practitioner. Webbed penis is one of the causes of inconspicuous or concealed penis according to the Maizels et al. [1] classification together with the buried penis, trapped penis, and the micro/diminutive penis. We think that webbed penis was missed at circumcision rather than secondary to it. Post-circumcision webbed penis differs from primary webbed penis. However, until now there has been no detailed classification for post-circumcision webbed penis. For that reason, the El Koutby and El Gohary [4] classification for primary webbed penis was used for grading the present studied patients. At the circumcision of a mild degree webbed penis, the operator should leave excessive ventral penile skin for web correction or downgrading of the webbed penis. On the contrary, the operator may excise more skin from the ventral part resulting in a higher grade of peno-scrotal web secondary to the deficient ventral skin. Moreover, the wide use of blind bone cutting forceps for circumcision, may add to these extreme entities. Classifying the circumcised webbed penis as a secondary type is far from what really exists. Webbed circumcised penis should have its own classification and grades. In the literature there are many studies that have described correction of the non-circumcised webbed penis. However, no studies have been described for circumcised cases. This may reflect a variation of circumcision and complication rates in each country. Many techniques are described to repair this anomaly in primary cases by simple or tailored excision of redundant skin with a linear scar that is liable to contracture [7]. For example, Dilley and Currie [8] used a technique of diamond-shaped marking for reconstructing the penoscrotal angle and avoids a vertical suture line crossing the peno-scrotal angle. Similarly, Alter [9] used single or double Z-plasty for the correction of webbed penis. He stated that ‘double Z-plasty causes less transverse scar length and little deformity. The angles of the Z-plasty are about 60 degrees, which give a theoretical length increase of 75%. All the sides of the “Z” are potentially equal in length’. A V-Y scrotoplasty [10] and double V-plasty modification are other options for the web repair. McLeod and Alpert [2] showed that they had, in their retrospective study, a 5% rate of skin wound separation in the ‘V’ scrotoplasty technique. This may explain the reported midline scar and even wound disruption, as a consequence of wound tension, in this technique. Chen et al. [11] described another technique for webbed penis correction in adults using a longitudinal median incision and the separation of the scrotal and ventral penile dartos with longitudinal closure. However, this technique corrects the web at the level of the dartos fascia only and does not resolve the problem if there is deficient ventral skin. The HM technique was developed by two surgeons at the end of 19th century for surgical correction of pyloric stenosis. It gives an additional length by converting the transverse incision to a longitudinal closure [3]. It has been used in other lengthening procedures in cases of tissue unavailability such as urethroplasty for urethral stricture [12]. Hanna and Bonitz [3] compared three different techniques: the HM scrotoplasty, V-Y scrotoplasty and Z-scrotoplasty to repair different grades of the non-circumcised webbed penis with acceptable results. They reported complications in 5.3% in the HM group, which is similar to our present study with a complication rate of 4.54% using the same technique. They also had 7.8% complications in the V-Y group and 2.9% in Z-plasty group. However, in our present study, only three (11.1%) patients developed self-limited penile oedema in Group II (multiple Z-plasty). We studied only two surgical techniques to be the standard for all the cases of post-circumcision penile web. The first one is the HM scrotoplasty for first-degree webbed penis. The second one is the Z-plasty technique in repairing second- and third-degree post-circumcision webbed penis. These two techniques repaired all the grades of post-circumcision penile web with few complications.

The present study had some limitations. The patients were grouped according to El Koutby and El Ghohary [4] classification for simple grouping of the patients and to propose a surgical correction of these cases with post-circumcision webbed penis. Post-circumcision webbed penis should have another detailed classification. We think that a longer follow-up for the corrected and uncorrected post-circumcision penile web should be considered especially in sexually active adults.

## Conclusion

The correction of various grades of post-circumcision webbed penis can be achieved by one of two techniques: the HM scrotoplasty in Grade 1 and the multiple Z-plasty in Grade 2 and 3, with favourable outcomes.

## References

[1] Maizels M, Zaontz M, Donovan J, et al. Surgical correction of the buried penis: description of a classification system and a technique to correct the disorder. J Urol. 1986;136:268–271. 2873259 10.1016/s0022-5347(17)44837-3

[2] McLeod DJ, Alpert SA. Double-V scrotoplasty for repair of congenital penoscrotal webbing: a hidden scar technique. J Pediatr Urol. 2014;10:810–814. 24572216 10.1016/j.jpurol.2014.01.014

[3] Hanna MK, Bonitz RP. Correction of congenital penoscrotal webbing in children: A retrospective review of three surgical techniques. J Pediatr Urol. 2016;12:161–165. 27020468 10.1016/j.jpurol.2016.02.003

[4] El Koutby M, El Gohary M. Webbed penis: A new classification. J Indian Assoc Pediatr Surg. 2010;15:50–52. 20975781 10.4103/0971-9261.70637 PMC2952775

[5] Weiss HA, Larke N, Halperin D, et al. complications of circumcision in male neonates, infants and children: a systematic review. BMC Urol. 2010;10:2. 20158883 10.1186/1471-2490-10-2 PMC2835667

[6] El-Sheemy MS, Ziada AM. Islam and circumcision. In: Bolnick DA, Koyle M, Yosha A, editors. Surgical Guide to Circumcision. London: Springer; 2012. p. 275–280.

[7] Masih BK, Brosman SA. Webbed penis. J Urol. 1974;111:690–692. 4363079 10.1016/s0022-5347(17)60048-x

[8] Dilley AV, Currie BG. Webbed penis. Pediatr Surg Int. 1999;15:447–448. 10415318 10.1007/s003830050631

[9] Alter GJ. Correction of penoscrotal Web. J Sex Med. 2007;4:844–847. 17627732 10.1111/j.1743-6109.2007.00512.x

[10] Chang SJ, Liu SP, Hseih JT. Correcting penoscotal web with V-Y advancement technique. J Sex Med. 2008;5:249–250. 17971100 10.1111/j.1743-6109.2007.00647.x

[11] Chen YB, Ding XF, Luo C, et al. A new plastic surgical technique for adult congenital webbed penis. J Zhejiang Univ Sci B. 2012;13:757–760. 22949367 10.1631/jzus.B1200117 PMC3437374

[12] Lumen N, Hoebeke P, Oosterlinck W. Ventral longitudinal stricturotomy and transversal closure: the Heinek-Mikulicz principle in urethroplasty. Urology. 2010;76:1478–1482. 20951413 10.1016/j.urology.2010.06.051

